# Phosphatidylethanol in Blood as a Marker of Chronic Alcohol Use: A Systematic Review and Meta-Analysis

**DOI:** 10.3390/ijms131114788

**Published:** 2012-11-13

**Authors:** Guido Viel, Rafael Boscolo-Berto, Giovanni Cecchetto, Paolo Fais, Alessandro Nalesso, Santo Davide Ferrara

**Affiliations:** Department of Molecular Medicine, Forensic Toxicology and Antidoping Unit, University of Padova, Via Falloppio 50, Padova 35121, Italy; E-Mails: giocecchetto@yahoo.it (G.C.); paolo.fais@yahoo.it (P.F.); alessandro.nalesso@unipd.it (A.N.); santo.davideferrara@unipd.it (S.D.F.)

**Keywords:** alcohol abuse, chronic excessive drinking, biological markers, phospholipids, phosphatidylethanol, mass spectrometry

## Abstract

The present paper aims at a systematic review of the current knowledge on phosphatidylethanol (PEth) in blood as a direct marker of chronic alcohol use and abuse. In March 2012, the search through “MeSH” and “free-text” protocols in the databases Medline/PubMed, SCOPUS, Web of Science, and Ovid/Embase, combining the terms phosphatidylethanol and alcohol, provided 444 records, 58 of which fulfilled the inclusion criteria and were used to summarize the current evidence on the formation, distribution and degradation of PEth in human blood: (1), the presence and distribution of different PEth molecular species (2), the most diffused analytical methods devoted to PEth identification and quantization (3), the clinical efficiency of total PEth quantification as a marker of chronic excessive drinking (4), and the potential utility of this marker for identifying binge drinking behaviors (5). Twelve papers were included in the meta-analysis and the mean (M) and 95% confidence interval (CI) of total PEth concentrations in social drinkers (DAI ≤ 60 g/die; *M* = 0.288 μM; CI 0.208–0.367 μM) and heavy drinkers (DAI > 60 g/die; *M* = 3.897 μM; CI 2.404–5.391 μM) were calculated. The present analysis demonstrates a good clinical efficiency of PEth for detecting chronic heavy drinking.

## 1. Introduction

Alcohol abuse and dependence are globally prevalent disorders, which span various socio-demographic groups and produce a broad range of secondary injuries and diseases [1,2].

Objective clinical and biochemical tests for characterizing the drinking pattern, quantifying the amount of daily ingested ethanol, and efficiently detecting alcohol-induced disorders are critically needed in both treatment and research areas [3,4].

Guidance on the investigation of suspected alcohol dependence or abuse includes symptoms, medical history, self-report forms, special questionnaires, clinical examination and biochemical investigations. Detailed efforts have been made to construct interview formats that correctly quantify alcohol intake, such as the “Alcohol Use Disorders Identification Test Consumption” (AUDIT-C) [5], the CAGE questionnaire [6], or which include reports from collateral individuals. These approaches exhibit, however, limitations in the forensic setting, where individuals are motivated to deny or minimize the magnitude of their drinking behavior in order to mitigate the professional and legal ramifications of alcohol abuse [7,8].

The limited diagnostic efficiency of self-reports and the difficulty in assessing alcohol-drinking behavior from an objective point of view have led in the last decades to an intensive search for reliable biomarkers of chronic excessive drinking; these markers can be broadly divided into direct and indirect categories. Indirect biomarkers detect the effects of alcohol on organ systems or body chemistry, and generally comprise markers of alcohol-related toxic effects, including mean corpuscular volume (MCV), aspartate aminotransferase (AST), alanine aminotransferase (ALT) and gamma-glutamyltransferase (GGT) [9,10]. More recent indirect markers examine ethanol-related biochemical changes in serum and comprise *N*-acetyl-beta-hexosaminidase (Beta-HEX), salsolinol, 5-hydroxytryptophol (5-HTOL), plasma sialic acid index of apolipoprotein J (SIJ) and the widely used carbohydrate deficient transferrin (CDT) [11,12]. Direct markers include blood ethanol itself, as well as alcohol derivatives, such as acetaldehyde, acetic acid, fatty acid ethyl esters (FAEE), ethylglucuronide (EtG), ethyl sulphate (EtS), and phosphatidylethanol (PEth) [13–16].

Among them, PEth in blood, and FAEE or EtG in hair, have attracted special attention as they are postulated to be highly specific and to roughly correlate with the ingested ethanol dose. Differently from FAEE and EtG, PEth in blood also seems to be promising for characterizing the drinking pattern (*i.e.*, identifying binge drinking episodes) and differentiating light-moderate drinking from abstinence.

For the above-mentioned reasons and given that all the identified review articles on the use of PEth in blood are based on descriptive data, a systematic review was conducted in order to summarize and better evaluate the diagnostic effectiveness of this marker in different clinical settings.

## 2. Results and Discussion

**Figure 1:**
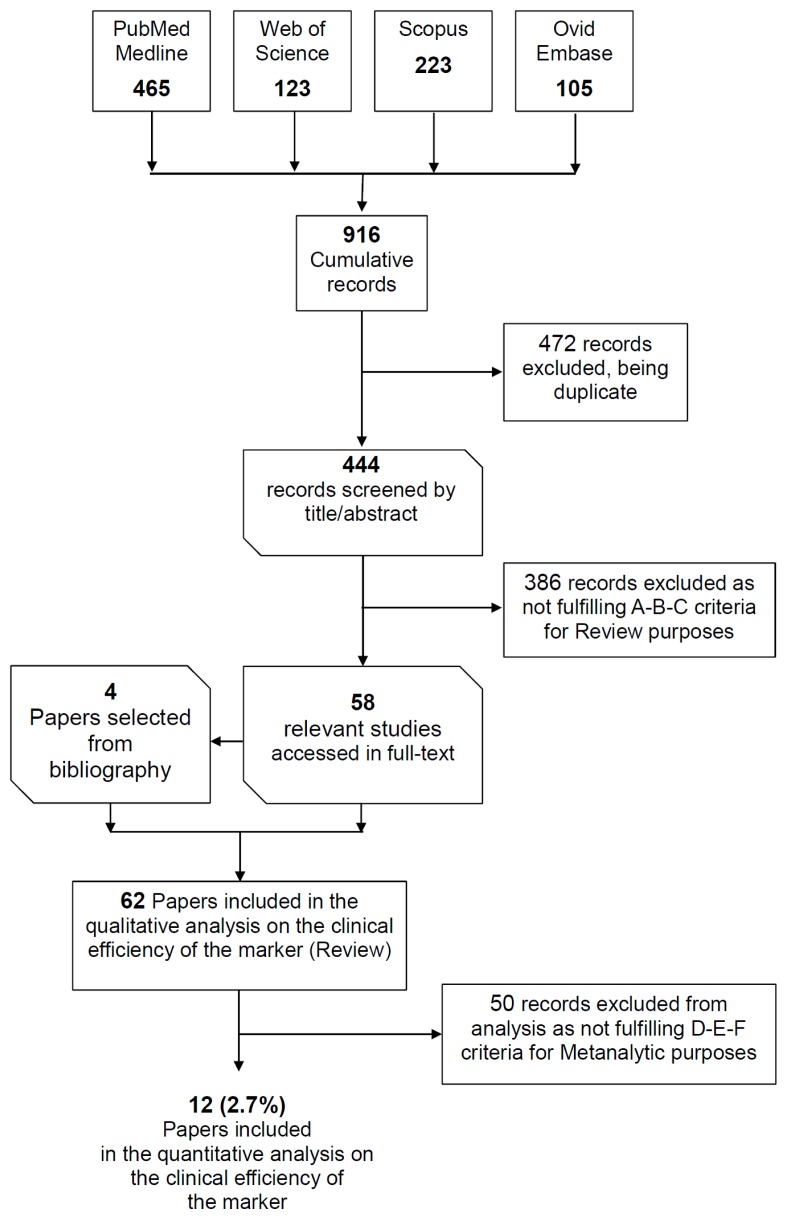
Search strategy and paper selection for inclusion in the systematic review and/or in the meta-analysis.

As reported in Figure 1, the combined search with both MeSH terms and free-text protocols in the databases PubMed, Web of Science, Scopus and Ovid/Embase retrieved 916 records, 472 of which were excluded, as they were duplicates. Of the 444 records screened by title and abstract, 386 were manually excluded, because they did not fulfill the inclusion criteria. In particular, in those manuscripts, PEth was used for characterizing the function and properties of the enzyme phospholipase D, not as a biomarker of chronic alcohol consumption.

Fifty-eight (58) potentially relevant papers were analyzed in full-text (Figure 1) and used to summarize the current evidence on:

– The formation, distribution and degradation of PEth in human blood (Section 2.1);
– The presence and distribution of different PEth molecular species in human blood (Section 2.2);
– The most diffused and efficient analytical methods devoted to the identification and quantisation of PEth in human blood (Section 2.3);
– The diagnostic efficiency of PEth as a clinical marker of chronic excessive drinking (Section 2.4);
– The potential utility of PEth as a marker of heavy episodic drinking or binge drinking (Section 2.5).

**Figure 2:**
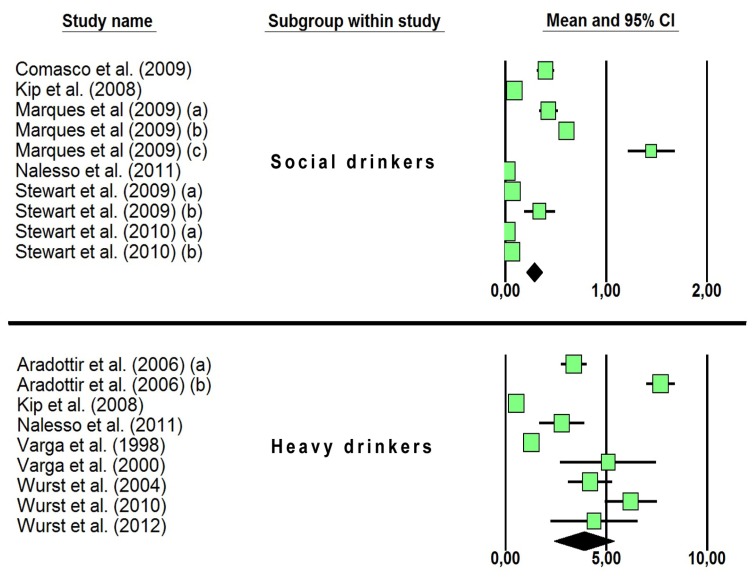
Schematic representation of the results of the meta-analysis performed on the 12 papers described in Tables 1 and 2. The investigated populations were classified based on daily alcohol intake (DAI): social drinkers (DAI ≤ 60 g/die), and heavy drinkers (DAI > 60 g/die). The black diamond represents the calculated mean and 95% CI for each subgroup. Lower case letters in brackets refer to multiple groups of subjects included in the selected studies (see Table 1).

Twelve (2.7%) studies were included in the meta-analysis (Tables 1 and 2; Figure 2) since they presented integrable data on PEth concentration in human blood, and thus on the diagnostic efficiency of the marker in detecting harmful drinking behaviors. The mean and 95% confidence interval (CI) of total PEth concentration in social drinkers (mean 0.288 μM; CI 0.208–0.367 μM) and heavy drinkers (mean 3.897 μM; CI 2.404–5.391 μM) were reported in Figure 2, and discussed in Sections 2.4 and 2.5.

### 2.1. Phosphatidylethanol Definition, Formation and Degradation

Phosphatidylethanol (PEth) is an abnormal cellular membrane phospholipid and was discovered for the first time in mammalians in 1983, being detected in the brain, kidney, liver, skeletal muscle, and heart of rats chronically exposed to ethanol [17–21].

#### 2.1.1. PEth Formation Mechanism

The formation of PEth is catalyzed by phospholipase D (PLD), an ubiquitary enzyme [20,22–24] normally devoted to the hydrolysis of phosphatidylcholine (PC) to phosphatidic acid (PA). PLD has a high affinity for short chain alcohols (100–1000-fold higher than for water). In the presence of ethanol, it promotes a transphosphatidylation reaction, with the production of PEth [23,25]. The expression of PLD varies dramatically among different animal species and also among the tissues of a single organism [26,27]. In humans, two different isoforms of the enzyme (PLD1 and PLD2), sharing 50% of the DNA sequence, have been genetically and functionally characterized. PLD1 has a perinuclear distribution and displays a very low basal activity, requiring a protein kinase C activation; PLD2 is localized in the cellular membrane and is constitutively active [28,29]. Both PLD1 and PLD2 catalyze the formation of PEth in human red blood cells [26–31].

#### 2.1.2. PEth Formation in Human Blood

It has been observed that the *in vitro* incubation of whole human blood with ethanol for 24 h (EtOH concentration varying between 50 and 100 mM) induces the formation of PEth, and that the EtOH concentration and the incubation time are directly proportional to the quantity of PEth produced. An activator of the protein kinase C added to the mixture triplicates the generated quantity of PEth [32]. No correlation between hematological indexes (red blood cell count, mean corpuscular volume, hematocrit) and the rate of PEth formation has been found [32].

Several studies on blood collected from mice, rat, ferret and pig have excluded the presence and the *in vitro* production of PEth in animal red blood cells (RBCs); thus, human RBCs seem to be peculiar in forming PEth *in vitro* in the presence of ethanol [32,33]. This characteristic represents an important drawback of PEth as a marker of chronic alcohol abuse: samples collected when the blood ethanol concentration (BAC) is higher than 0.1 g/L can generate false positive results due to the neo-formation of PEth *in vitro* (in the post-sampling period), which can occur at room temperature (16 °C–20 °C), but also at −20 °C, being slower at +4 °C [32]. Only at −80°C can the formation process be considered drastically inhibited [32–34].

#### 2.1.3. PEth Degradation Mechanism

Another important and peculiar characteristic of human RBCs is the incapacity of efficiently degrading PEth, probably due to the absence of phosphatidylcholine phospholipase C (PLC) activity [32–35]. PEth elimination, with a half-life (*t*/2) varying between 0.5 and 2 h, has been demonstrated in several human cellular systems, such as pancreatic islets [36], hepatocytes (HepG2 and C6 cells) [36–38], and neutrophils, but not in RBCs [32,38]. The molecular mechanism of PEth elimination has not yet been fully elucidated; although phospholipase A2 and PLC *in vitro* release arachidonic and palmitic acid from PEth [20,39], there is still a lack of evidence that phospholipases are involved in the *in vivo* PEth degradation. What is clear is that in human RBCs there is a disproportion between PEth formation and degradation rates, causing PEth to accumulate in the cellular membranes, an accumulation that suggests a potential use of PEth for detecting chronic exposure to ethanol.

#### 2.1.4. PEth Degradation in Human Blood

In clinical studies conducted on chronic heavy drinkers, PEth was found to be detectable in blood up to 28 days after sobriety [32,40–46]. In 15 alcoholics following a detoxification program, the mean half-life of blood PEth was 4.0 ± 0.7 days with a range of 3.0–5.3 days [47]. The kinetics of elimination was well-approximated by a one-compartment model. More recently, this degradation kinetics has been confirmed on 57 alcohol-dependent subjects following a detoxification program [45]. PEth decreased over time with a half-life of about 3–5 days and was detectable in 64.3% of the cases after 28 days of sobriety [45]. Additionally, it has been demonstrated that sex, gender, age and body mass index do not influence the normalization rate of PEth [45].

In a recent experiment during which, after three weeks of abstinence, 11 social drinkers were exposed to an amount of ethanol of 1 g/Kg for five consecutive days (daily alcohol intake ranging between 67 and 109 g/die), and then remained abstinent for 16 days, undergoing regular and scheduled blood sampling, the mean half-life of PEth ranged from 4.5 to 10.1 days in the first week and from 5.0 to 12.0 days in the second week [48].

### 2.2. Phosphatidylethanol Molecular Species

Since the very early studies conducted on rats chronically exposed to ethanol [17–22,49] it has been observed that PEth is not a single molecule, but a group of glycerophsopholipid homologues with a common phosphoethanol head group onto which two long carboxylic acid side chains, typically containing from 14 to 22 carbon atoms with different grades of insaturation (0–6 double bonds), are attached [50–52].

These homologues are commonly named in the form “PEth A:B/C:D” where A and C indicate the number of carbons in the carboxylic side chains, whereas B and D indicate the number of double bonds in each side chain [34].

Fast atom bombardment-mass spectrometry experiments on PEth, formed after bradykinin or phorbol ester PLD stimulation in pheocromocytoma cells (PC12), revealed that the molecular species of the generated PEth were almost identical to those of PC, consistent with this lipid being the substrate of PLD [51]. Additionally, using neuroblastoma cells (NG 108-15) it has been demonstrated that the addition of polyunsaturated fatty acids to the medium induced similar changes in the fatty acid composition of PC and PEth [52,53].

In one of the studies by Alling *et al.*[17] on the distribution of PEth in the organs of rats chronically treated with ethanol, significant inter-organ differences in the fatty acid composition of PEth have been highlighted: brain PEth contained high proportions of palmitic (16:0) (28%) and oleic (18:1) (34%) acids with a 10% of polyunsaturated fatty acids (mainly arachidonic acid, 20:4), whereas liver PEth contained more stearic acids (18:0) and less palmitic acids (16:0) [17].

Only in recent times was the characterization of PEth molecular species in human blood performed, through highly sensitive liquid chromatography multiple mass spectrometry methods (LC-MS/MS) [50,54–57]. In blood collected from heavy drinkers PEth 16:0/18:1 and 16:0/18:2 have been demonstrated to be the predominant molecular species accounting on average for 37%–46% and 26%–28%, respectively, of total PEth in blood [50,57]. Owing to inter-individual variations, PEth 16:0/18:2 was sometimes the major form, whereas PEth 16:0/20:4 constituted about 8%–13% of total PEth, and PEth 18:1/18:1 and 18:0/18:2 taken together constituted about 11%–12% of total PEth. The species 16:0/16:0, 16:0/20:3, 18:0/18:1, 16:0/20:3 and 18:1/18:2 had a lower blood concentration, each one accounting for about 1%–5% of total PEth [50,57]. Diet was suggested to be the main factor determining the fatty acid pattern in PC and PEth, although the influence of genetic determinants, drinking pattern and metabolic disorders needs to be further clarified.

The numerous combinations of chain length and double bonds enable the formation of a very large theoretical number of different PEth species and so far 48 homologues have been identified in post-mortem human blood [55]. Although inter-individual differences in the relative abundances of PEth homologues do exist, the preliminary data available suggest that 5 molecular species (16:0/18:1, 16:0/18:2, 16:0/20:4, 18:1/18:1, 18:1/18:2) could constitute more than 80% of total PEth.

### 2.3. Analytical Techniques and Methods for PEth Identification and Quantization in Blood

For PEth determination, venous blood should be collected in tubes containing ethylenediamine tetra-acetic acid (EDTA) and the sample should not be centrifuged [34]. Blood samples for PEth analysis have proved to be stable for 24 h at room temperature and for 3 weeks at +4 °C [32,58]. For longer periods of storage, whole blood should be frozen in a plastic tube and kept at −80 °C, to avoid any *in vitro* formation [34].

Several analytical strategies have been utilized so far for quantifying total PEth concentration in blood, mainly based on chromatographic or electrophoretic separation methods, as reported below.

– Thin layer chromatography (TLC) [17–20,59].
– High performance liquid chromatography (HPLC) coupled to an evaporative light-scattering detector (ELSD) [34,47,60–62].
– Gas-chromatography coupled to mass spectrometry [63].
– Non-aqueous capillary electrophoresis (NACE) [64,65].
– Immunoassay with PEth-specific monoclonal antibodies [66,67].

Thin layer chromatography (TLC) is a manual semi-quantitative method with limited sensitivity and throughput and was the very first method used for identifying PEth in animal tissues [17,18], quickly judged unsuitable for further experimental studies. The first and only gas-chromatographic method reported in the literature was developed for the detection of two derivatization products of PEth, ethyl bis (trimethyl-silyl)-phosphate and tris (trimethyl-silyl)-phosphate; probably due to a lack of specificity of the monitored derivatization products, this method has never been applied to the analysis of clinical samples [63].

Similarly, to the best of our knowledge, the immunochemical method based on the anti-PEth antibodies 2B1 e 2E9 generated with the traditional hybridoma technique, capable of detecting *in vitro* formed PEth, has not yet found a clinical application [66,67].

The two electrophoretic methods based on non-aqueous capillary electrophoresis, either coupled to an UV [64] or to a mass spectrometric detector [65] have also found very limited clinical application.

On the contrary, the HPLC-ELSD method is actually the most utilized in clinical toxicology. Briefly, whole blood together with the internal standard (phosphatidylbutanol 18:1/18:1) are extracted with 2-propanol and hexane, followed by the quantification of PEth in the extract on an HPLC system equipped with an evaporative light scattering detector (ELSD) and with PEth 18:1/18:1 as calibrator [47,60–62].

More recently several liquid chromatography mass spectrometry (LC-MS) or multiple mass spectrometry methods (LC-MS/MS) have been developed for the identification of PEth homologues in blood, the majority of which employ electrospray ionization (ESI) for MS coupling [50,55–58], although time-of-flight (TOF) mass spectrometry has also been proposed [68]. Also a LC-MS/MS method for the analysis of PEth 16:0/18:1 and 18:1/18:1 on dried blood spots, which exhibited a good correlation with parallel determinations on fresh blood, has been developed and validated [69].

All these novel mass spectrometric methods exhibit a considerably higher analytical sensitivity (more than two orders of magnitude) and shorter turnaround time with respect to HPLC-ELSD, and are capable of identifying PEth species also in blood collected from social drinkers [57,70–73].

A considerable limitation of the above-mentioned methods, however, is the absence of commercially available reference substances for PEth analogs (at the moment only PEth 16:0/16:0, 16:0/18:1 and 18:1/18:1 are available), complicating the validation process, and hindering the diffusion of these methods in clinical and forensic toxicology laboratories. Zheng and coworkers have solved the problem by preparing in house deuterium-labeled PEth analogs for PEth 16:0/18:1 and PEth 16:0/18:2. This commendable effort is however too complex and time-consuming to leave the research arena and enter routine laboratory practice [58].

### 2.4. Diagnostic Efficiency of PEth as a Clinical Marker of Chronic Excessive Drinking

Four out of twelve articles included in the present meta-analysis are clinical trials involving inpatients with a diagnosis of alcohol-dependence based on the Diagnostic and Statistical Manual IV-R (DSM IV-R) or the International Classification of Diseases (ICD-10) criteria, who underwent a detoxification program (see Table 1). These studies are generally aimed at evaluating the diagnostic efficiency of PEth in comparison to clinical interviews, questionnaires and/or other traditional markers of harmful drinking, and at correlating the concentration of PEth to the amount of ethanol ingested in the previous 2–4 weeks [40,43–47,57,70,72–77]. Only one of those studies used an intergroup control [57], the rest being uncontrolled trials.

The concentrations of total PEth in alcohol dependent subjects admitted for detoxification vary significantly in the considered clinical studies (range: 0.0–7.7 μM; see Table 2). With respect to the mean values and 95% CI of PEth concentrations reported in the meta-analyzed papers (see Table 2 and Figure 2), the heavy drinkers group (Daily Alcohol Intake—DAI > 60 g) is well separated from the social drinkers, displaying a mean concentration one order of magnitude higher than the remaining groups (heavy drinkers = 3.897 μM; social drinkers = 0.288 μM).

At the present time, the international scientific community has not yet established a cut-off value for PEth concentration in blood to be used for differentiating an acceptable social ethanol intake (<40 g for males and <20 g for females, according to the World Health Organization parameters), from an at-risk-alcohol-use (40–60 g/die) and chronic excessive drinking behavior (>60 g/die).

Nine of the above-mentioned clinical trials used HPLC-ELSD for total PEth quantification in blood utilizing as an interpretative cut-off the lower limit of quantification (LOQ) of the analytical method used for PEth determination, that is: 0.22 μM [40,46,47,70,77], 0.30 μM [44,45,75] or 0.8 μM [43]. With the above-mentioned cut-offs, the diagnostic sensitivities of PEth were very high, varying from 98% [40] to 100% [44–46,57].

In Sweden, 0.7 μM is currently used as the routine clinical threshold [50], although that value has not yet been approved at a supranational level [78].

Regarding clinical specificity in differentiating alcohol-dependent subjects from social drinkers and/or abstainers, the available controlled trials have all obtained a 100% value [42,57,73]. This absolute specificity of the biomarker can only be partly explained by the intrinsic characteristics of PEth, which is formed in blood only in the presence of ethanol. Potential bias of selection have to be considered, due to the fact that the case-control populations considered exhibited significant differences in the drinking pattern and in the amount of daily-ingested ethanol (see Table 1).

Differing from the traditional indirect biomarkers used for diagnosing a chronic excessive drinking behavior (MCV, AST, ALT, GGT, and CDT), blood PEth concentration seems not to be influenced by age, gender, other ingested substances or non alcohol-associated diseases, such as hypertension, kidney and/or liver diseases [44–46,72]. For these reasons, PEth is considered to perform better than MCV and GGT, both in terms of sensitivity and specificity, for detecting chronic excessive drinking behaviors [40–43,45,46]. A few studies have also highlighted a slightly higher sensitivity of PEth, compared to the most selective indirect marker CDT [40,42,43].

Preliminary evidence does exist concerning a rough correlation between the amount of ethanol consumed in the previous two weeks and the concentration of PEth in blood [40,44,45,57], although observations of individual PEth formation rates [32] and recent studies on moderate drinkers indicate that it might not always be possible to link PEth concentration in blood to a precise drinking level [72,73].

### 2.5. PEth as a Potential Marker of Heavy Episodic Drinking or “Binge Drinking”

An especially interesting question, which still needs to be answered, regards the quantity of ethanol that must be consumed for a certain time-period to give a positive blood PEth assay. Using the HPLC-ELSD method of analysis, it has been observed that a single ethanol dose of 30–47 g did not produce any measurable amounts of PEth in blood [43]. The threshold of total ethanol intake leading to a positive PEth assay was estimated at around 1000 g in three weeks, with a daily consumption of at least 50 g/die [43,45]. A recent drinking experiment, which employed a more sensitive LC-MS/MS method for the quantification of PEth 16:0/18:1, conducted on 11 healthy volunteers who drank 50–109 g of ethanol/die, showed that the formation of PEth began immediately after the first assumption of alcohol (0.5–8 h) reaching a concentration of about 0.05–0.10 μM and then stagnated or decreased when the blood alcohol concentration started to decline [48]. These results are certainly encouraging because they pave the way for novel potential applications of PEth in the diagnosis of excessive drinking episodes and/or “binge drinking” behaviors; at the same time, they do underline the compelling need to study large populations of social drinkers and teetotalers in order to determine whether an efficient cut-off can be established for differentiating teetotalers from social/moderate drinkers and “binge drinkers”.

At the present time, only five studies have examined the characteristics of total PEth as a potential marker of “binge drinking” [72,73,75–77]; the small number of the investigated subjects, the variability of the clinical methods used to reconstruct the daily alcohol intake (see Table 1), and the diversity of the analytical methods employed for quantifying total PEth in blood (see Table 2) has not yet allowed any definitive conclusion to be drawn. Comasco *et al.*[75] have found a sensitivity of only 9% for detecting a moderate alcohol consumption (>2 drinks/week) in 200 adolescent students from Vestmanland, with the limited clinical efficiency of PEth probably explained by the low sensitivity of the analytical method (HPLC-ELSD) and the subject stratification employed.

Stewart and colleagues [73], examining 80 healthy women in reproductive age, have found that a PEth 16:0/18:1 concentration above 0.18 μM was highly specific for identifying women drinking more than 28 g per day, although a relevant degree of inter-individual variability was evident; this variability could only partly be explained by the different timing of assumption (with respect to blood sampling) and the different drinking patterns (regular moderate intake *vs.* episodic heavy drinking) [73]. Although being expensive, we do believe that only controlled drinking experiments over a period of 2–3 weeks could highlight if and how PEth could be utilized as a marker of “binge drinking” or as a marker of absolute abstinence.

## 3. Experimental Section

### 3.1. Search Strategy

In March 2012, one of the authors (GV) performed the systematic search of the literature searching Medline/PubMed, SCOPUS, Web of Science, and Ovid/Embase databases. The Medline search employed a complex search strategy including both “MeSH” and “free-text” protocols. More specifically, the following terms retrieved from the MeSH browser provided by Medline were utilized: (“Ethanol” [MeSH] OR “Alcohol Drinking” [MeSH]) AND “Phosphatidylethanol” [MeSH]. A multiple “free-text” search restricted to the fields “title/abstract” was performed combining by “AND” the entry terms “Alcohol” and “Phosphatidylethanol”. No temporal limits were utilized. Because of the interface limitation only the “free-text” protocol “((Phosphatidylethanol OR PEth) AND (Ethanol OR Alcohol))” was used for searches in Ovid/Embase, Web of Science (search field: “topic”), and SCOPUS (search fields: title/abstract, keywords).

### 3.2. Paper Selection

Paper selection was conducted independently by three reviewers (GV, RBB, AN), based on titles and abstracts of papers retrieved by the systematic search. The following inclusion and exclusion criteria were adopted for review and meta-analytic purposes. Any discrepancy in the paper selection and data extraction was settled by consensus discussion.

#### 3.2.1. Inclusion criteria for review purposes

To meet the inclusion criteria for the present review, studies had to fulfill at least one of the following requirements:

- Provide data on PEth concentration in human blood collected from alcohol dependent subjects, heavy drinkers, moderate drinkers, social drinkers or teetotalers.
- Provide data on PEth concentration in fluids or tissues of animals chronically exposed to ethanol.
- Describe an analytical method for the quantification of total PEth or PEth molecular species in human blood.

#### 3.2.2. Exclusion criteria for review purposes

Articles not fulfilling at least one of the previous requirements or characterizing the activity of phospholipase D (PLD) were excluded.

In the case of doubtful classification based on solely title and abstract, the full text was retrieved. Whenever this was ineffective, the question was settled by consensus discussion.

#### 3.2.3. Inclusion criteria for meta-analytic purposes

To meet the inclusion criteria for the present meta-analysis, studies had to fulfill all the following requirements:

(D) Fulfilling criterion A for Review purposes.
(E) Reporting integrable data on blood concentrations of PEth in humans.
(F) Reporting integrable data on the daily alcohol intake (DAI) of the subjects recruited in the study.

#### 3.2.4. Exclusion criteria for meta-analytic purposes

Articles not fulfilling all of the above-mentioned requirements, being commenting letters or reviews, reporting data not comparable/suitable for direct meta-analysis processing (*i.e.*, inappropriate statistical formats) or not amenable by extraction or calculation/conversion from the published results or figures, were excluded.

### 3.3. Data Extraction

Data extraction was conducted independently by four authors (GV, RBB, AN, PF) and the data derived from the studies were collected in an electronic database, while two different authors (GC, SDF) verified the accuracy of the data extraction process, in order to minimize subjective judgment. The following items were collected from each study: authors, publication year, features of the study (main aims, inclusion and exclusion criteria, duration of the follow-up), characteristics of the investigated population (numbers of subjects, age, race, comorbidities, use of medications or assumption of illicit drugs, clinical setting, type of controls used), type and amount of alcohol consumption (estimation of alcohol use, daily mean ingested ethanol, type of stratification if any, timing of sample collection, biological assessments before collection), analytical methods used for PEth analysis, type of measured PEth and concentrations, type of collected sample, clinical efficiency of PEth (sensitivity, specificity, positive predictive value, negative predictive value), and other markers used for detecting chronic excessive drinking. Any discrepancy in data extraction was settled by consensus discussion.

In order to meta-analyze the distribution of PEth concentration with regard to the DAI, data were grouped into two categories, as follows, considering the mean or median of DAI for the classification of the drinking behavior.

Category 1. Social drinkers (DAI ≤ 60 g).
Category 2. Heavy drinkers (DAI > 60 g).

Specifically, these categories were overlapping for some extreme values (ranging from 40 to 60 g), which represented the mathematical limits of the Standard Deviation or the Interquartile Range. This reflected the lacking of a shared threshold to discriminate DAI among groups. Nevertheless, the descriptive statistics of DAI also reported the most important indexes of central tendency (mean and median), which were markedly different between groups (Table 2), hence not impairing our analysis.

### 3.4. Statistical Analysis

One of the authors (RBB) performed the meta-analysis according to a previously published procedure [79,80]. Statistical analyses of continuous variables were performed using the weighted mean as the summary statistic reported with 95% confidence intervals. To allow inference to an external population, a random-effects model was used for purposes of meta-analysis in a conservative setting [79,81].

## 4. Conclusions

The present systematic review demonstrates that total phosphatidylethanol, an abnormal phospholipid formed in the erythrocyte membrane exclusively in the presence of ethanol, exhibits high diagnostic sensitivity and specificity for detecting active chronic excessive drinking behaviors, with a regular daily alcohol intake (DAI) of more than 60 g.

The mean values and confidence intervals of total PEth concentrations in blood of heavy (DAI > 60 g/die) and social drinkers (DAI ≤ 60 g/die) showed a significant statistical difference. These findings demonstrate a good clinical efficiency of PEth for detecting heavy drinking.

The recent introduction of sensitive analytical methods based on liquid chromatography coupled to mass spectrometry detection, capable of effectively measuring single molecular species of PEth in blood in the nanomolar range, has opened promising new application fields for PEth, such as the identification of minute alcohol consumption (*i.e.*, monitoring of abstinence), and the identification of heavy episodic drinking behaviors (*i.e.*, “binge drinking”). However, large randomized trials are needed in order to ascertain if PEth is really effective in those diagnostic challenges.

Moreover, it is essential to set up international standards that properly define the characteristics of the alcohol biomarker PEth and harmonize the methodology used in both clinical and laboratory investigations. The following five issues certainly need to be addressed.

1. Define the molecular species of PEth that should be determined in blood when a mass spectrometric analytical method is used; it needs to be clarified if PEth 16:0/18:1 and 16:0/18:2 (the two most abundant homologues) are sufficient to resemble the total PEth concentration or whether it would be more appropriate to investigate and quantify multiple PEth molecular species; the data derived from our analysis suggest that at least 5 molecular species (PEth 16:0/18:1, 16:0/18:2, 16:0/20:4, 18:1/18:1, 18:1/18:2) could be used.
2. Develop and market commercial reference substances for the PEth molecular species identified by point 1.
3. Arrive at a consensus on the most appropriate cut-off for differentiating social alcohol use from heavy drinking.
4. Characterize the kinetics of formation and degradation of the identified PEth species in order to determine whether they might be used for monitoring abstinence.
5. Verify the correlation of blood PEth concentration with the amount of ethanol ingested in the previous two weeks.

## Figures and Tables

**Table 1:** The features of the 12 selected papers (study, year, main aim of interest, inclusion and exclusion criteria, and duration of the follow up) and the investigated populations (number of subjects, mean age, race, comorbidities, clinical setting, subject stratification, and type of controls used) are here summarized.

Study	Year	Features of the study				Investigated population						
		Main aim of interest	Inclusion criteria	Exclusion criteria	Duration of follow-up	Number of Subjects	Mean Age (Ys) *	Race	Comorbidities	Clinical setting	Subjects stratification	Type of controls
Aradottir *et al.*	2006	Diagnostic sensitivity of PEth and correlation to ethanol consumption (last 14 days)	Diagnosis of alcohol dependence based on DSM IV and ICD-10	BrAC < 0.1 g/L Illicit Drugs Severe medical illness	-	66 actively drinking patients attending a programme of consultation for problematic drinking (55 M/11 F)	49.1 ± 9.9	-	-	Outpatients Inpatients	By timeline follow-back: “Low” < 40 g (*n* = 10)	-
											“Moderate” 40 to 80 g (*n* = 28)	
											“High” 80 to 200 g (*n* = 60)	
						78 patients admitted to a detoxification unit (68 M/10 F)	52.9 ± 8.5				“Very High” > 200 g (*n* = 43)	
Comasco *et al.*	2009	Comparison of diagnostic efficiency of PEth to clinical interview in detecting high alcohol consumers	Students with deviant behaviour as reported by the Survey of Adolescent Life in Vestmanland	-	-	200 adolescent students (57 M/78 F)	-	-	-	Outpatients	By semistructured interview: Low alcohol consumption (*n* = 96): consuming alcohol < 2/mo and never, seldomly, or occasionally became intoxicated, or intermediate frequency alcohol consumers who never or seldomly became intoxicated High alcohol consumption (*n* = 104) are “cases”: consuming alcohol ≥ 2/mo and always or almost always became drunk	-
Kip *et al.*	2008	Investigate the diagnostic performance of PEth	Negative BAC	Age < 18 y BAC > 0.1g/L Recent use of illicit drugs, Pain ≥ 3 on Visual Analogue Scale, Liver cirrhosis (Child B or C) and renal diseases, Mental illness, non-fluency of German language, police custody or inability to give informed consent	-	52 Male patients presented at the Emergency room with angina pectoris (ICD 10 I20) or gastrointestinal complaints (ICD 10 K92.9) AUDIT < 8 (*n* = 52)	61 (IQR 39–66)	-	Smokers 28.8%	Outpatients	By AUDIT: AUDIT < 8 (*n* = 52)	-
						22 Male patients presented at the Emergency room with angina pectoris (ICD 10 I20) or gastrointestinal complaints (ICD 10 K92.9) AUDIT ≥ 8 (*n* = 22)	52 (IQR 38–64)		Smokers 50%		AUDIT ≥ 8 (*n* = 22)	
Nalesso *et al.*	2011	Correlate PEth to self-reports on alcohol assumption	-	BAC < 0.1 g/L	-	11 Heavy drinkers admitted to a detoxification unit (7 M/4 F)	49 (IQR 37–57)#	-	-	Inpatients	-	Intergroup
						8 Social drinkers (5 M/3 F)	42 (IQR 32–56)#			Outpatients		
						10 Teetotallers (6 M/4 F)	32.5 (IQR 27–39)#			Outpatients		
Stewart *et al.*	2009	Evaluate the relationship between PEth and recent drinking in patients with liver disease and hypertension	Recent drinking	Cognitive dysfunction precluding informed consent Abstainers	-	21 Liver disease patients (13 M/8 F)	50 (33–64)$	6 Hispanic white 15 non-Hispanic white	21 Liver disease with Model for End-Stage Liver disease 16 (6–32)$; 5 with chronic Hepatitis C	Inpatients and Outpatients	By average drinks per day (each drink = 14 g): <14 gr (*n* = 17)	-
											(14 ≤ *x* ≤ 42) g (*n* = 14)	
						21 Hypertension patients (15 M/6 F)	60 (44–74)$	12 Hispanic white 9 non-Hispanic white	-		≥ 42 g (*n* = 11)	
Varga *et al.*	1998	Investigate PEth levels after a limited ethanol intake	Abstainers or “small amount” alcohol consumers	-	21 days	17 Population with no or limited alcohol intake (11 M/6 F)	25–47$	-	-	Outpatients	5 abstainers (3 M/2 F)	-
							19–31$				12 social drinkers (8 M/4 F)	
Stewart *et al.*	2010	Evaluate the relationship between blood PEth and alcohol use in reproductive age women	Generally healthy women Age 18–35 ys Self-reported consumption of any amount on at least two days/wk	Pregnant women and abstainers	-	80 healthy women (80 F)	26 (IQR 23–30)	71 Non-Hispanic white 6 Non-Hispanic-black 3 Others	-	Outpatients	By average drinks per day >1 (14 g) (*n* = 64)	-
											>2 (28 g) (*n* = 28)	
											All the cases	
Wurst *et al.*[45]	2010	Determine the correlation of PEth to self-reports	Alcohol dependent detoxification patients (ICD 10 F10.25)	Severe liver, renal and brain diseases, metabolic disorders, intake of illicit drugs, BAC < 0.1 g/L	28 days	57 alcohol dependent detoxification patients (48 M/9 F)	43.6 ± 10.4	-	-	Inpatients	-	-
Wurst *et al.*	2004	Evaluate the effect of using a low cut-off to identify heavy drinking/alcohol dependence by PEth in whole blood	Meeting ICD 10 criteria for alcohol-dependence	-	-	18 detoxification patients (14 M/4 F)	44 (24–55)$	-	Smoked cigarettes per day: 20 ± 12.6	Inpatients	-	-
Wurst *et al.*	2012	Explore Sensitivity and Specificity of PEth	Meeting ICD 10 F10.25 criteria	-	28 days	5 alcohol dependent patients (5 M/0 F)	40 (IQR 36–58)#	-	-	Inpatients	-	-
Marques *et al.*	2009	Identify alcohol biomarkers related to driver’s BAC patterns from IIDs	With IIDs (ignition lock at 0.04 g/dL)	-	8 months	534 DUI offenders (464 M/70 F): 208 alcohol dependent 64 alcohol abusers	38.7 ± 11.5	91% Caucasian 9% Others	-	Outpatients	By fail rates at interlock BrAC test: 0 lockouts (*n* = 136)	-
											0 < lockouts ≤ 1.45% (*n* = 268)	
											> 1.45% lockouts (*n* = 104)	
Varga *et al.*	2000	Investigate elimination kinetics of PEth	Chronic alcoholics admitted to a detoxification unit	-	-	6 Chronic alcoholics (6 M/0 F)	-	-	-	Inpatients	-	-
					7 days	15 Chronic alcoholics (13 M/2 F)						

* Data are reported as Mean (M) ± Standard Deviation (SD), or Median (Me) with Interquartile Range (IQR) according to the type of statistical distribution;

- = Not reported;

$ Total range is reported;

# Calculated from the reported data;

wk = week/s; mo = month/s; ys = years; BAC = Blood Alcohol Concentration; BrAC = Breath Alcohol Concentration; IIDs =Ignition Interlock Devices; DUI = Driving Under the Influence of alcohol; DSM-IV = Diagnostic and Statistical Manual of Mental Disorders IV; AUDIT = Alcohol Use Disorders Identification Test; SRC = Self-Reported Consumption; PEth = Phosphatidil-Ethanol.

**Table 2:** Data on the frequency and amount of alcohol consumption of the subjects recruited in the 13 selected papers (methods for estimating alcohol use, mean daily alcohol consumption before test, timing of blood sampling and analysis), the analytical method used, the mean blood concentrations of total PEth, and the diagnostic efficiency of the marker (sensitivity, specificity, positive and/or negative predictive value) are presented.

Study	Year	Alcohol assumption				PEth Determination								
		Methods for estimating alcohol use	Daily Mean Alcohol consumption before test (g/die)	Timing of blood retrieval	Biological assessment before blood retrieval	Type of sample	Form of Measured PEth	Analytical method LOQ # Cut-off **	Concentration μM	Sensitivity	Specificity	PPV	NPV	Other markers
Aradottir *et al.*	2006	Timeline follow-back (14 days)	103 ± 64 Outpatients 204 ± 126 Inpatients	Single retrieval	Negative BrAC within 10 previous hours	Whole Blood	Total PEth	HPLC-ELSD 0.22 μM #**	3.4 ± 2.6 Outpatients 7.7 ± 3.2 Inpatients	98% Outpatients 100% Inpatients	-	-	-	%CDT GGT MCV
			0–40$							100%°	-	-	-	
			40–80$							96.9%°				
			80–200$							100%°				
			>200$							100%°				
Comasco *et al.*	2009	Survey of adolescent life in Vestmanland (1 last year) Semi-structured interview	*M* = 6.2° *F* = 5.5° (daily mean during the last year)	Single retrieval	-	Whole Blood	Total PEth	HPLC-ELSD 0.30 μM #**	High alcohol consumers testing positive (*n* = 9) 0.4 (0.25–0.71)&$	9%	96%	69%	49%	FAEE
Varga *et al.*	1998	Self Reported Consumption (3 weeks)	47 g (M) or 32 g (F) (*n* = 5)	Hours: 0.5 – 1 – 2 – 4	-	Whole blood	Total PEth	HPLC-ELSD 0.8 μM #**	-	-	100%	-	-	CDT, GGT
				Days: 1					<LOQ					
				3					<LOQ					
				5					<LOQ					
			63.5 ± 25.3 (*n* = 12)	Days: 1					<LOQ					
				18					1.4 ± 0.6°					
				21					1.3 ± 0.5°					
Kip *et al.*	2008	AUDIT Self Reported Consumption (1 week)	20 (IQR 0–43)	Single retrieval	Negative BAC	Whole blood	Total PEth	HPLC-ELSD 0.22 μM #	0.0 (IQR 0.0–0.35)	-	-	-	-	%CDT GGT MCV EtG in serum and urine
			60 (IQR 43–100)						0.33 (IQR 0.0–1.49)					
Nalesso *et al.*	2011	Self Reported Consumption (1 week)^	10 (IQR 9–15)°	Single retrieval	Negative BAC	Whole blood	17 PEth molecular species	LC/HRMS 0.001 μM #	2.8 ± 1.9°	100%	-	-	-	-
			9 (IQR 4.5–13.5)°						0.021 ± 0.033°					
			0°						<LLOQ°					
Stewart *et al.*	2009	Self Reported Consumption (2 weeks)	11.8 (IQR 0–51.8)°	Single retrieval	-	Whole blood	16:0/18:1	LC/MS-MS 0.03 μM #	0.06 (IQR 0–0.06)°	-	-	-	-	-
			26.7 (IQR 10.1–38.5)°						0.14 (IQR 0.08–0.29)°					
									0.98 (IQR 0.47–1.71)°					
Stewart *et al.*	2010	Self Reported Consumption (2 weeks)	25.4 (IQR 14.6–98.4)°	Single retrieval	-	Whole blood	16:0/18:1	LC/MS-MS 0.03 μM #	<14 g/day 0.02 ± 0.04°	61%	95%	-	-	-
			44.2 (IQR 28.4–98.4)°						(14 < X < 28) g/day 0.07 ± 0.08°	32%	95%			
			23 (IQR 15.1–38.1)°						>28 g/day 0.19 ± 0.18°	-	-			
Wurst *et al.*	2010	Timeline follow-back (1 week) or AUDIT	207.4 ± 115.9	Days: 1	-	Whole blood	Total PEth	HPLC-ELSD 0.30 μM #**	4.7 ± 4.97	100%	-	-	-	%CDT GGT MCV
				3					3.09 (IQR 0.81–5.15)°&	93.7%				
				5					2.42 (IQR 0.78–4.24)°&	94.4%				
				7					1.69 (IQR 0.55–2.9)°&	94.1%				
				14					0.88 (IQR 0.18–1.69)°&	66.7%				
				28					0.81 (IQR 0.0–2.12)°&	25%				
Wurst *et al.*	2004	Timeline follow-back (1 month)	148 (32–253.3)$°	Single retrieval	-	Whole blood	Total PEth	HPLC-ELSD 0.30 μM #**	0.0037 (IQR 0.00063–0.00868)°	100%	-	-	-	FAEE
Wurst *et al.*	2012	Self Reported Consumption (1 week) AUDIT	240 (IQR 160–352)°	Days: 1	-	Whole blood	Total PEth	HPLC-ELSD 0.22 μM #**	4.40 ± 2.45°	100%	-	-	-	MCV, GGT, SIJ, UEtG, UEtS
				3										
				7					2.34 ± 1.57°					
				10										
				14					1.28 ± 0.67°					
				21					0.77 ± 0.35°					
				28					0.36 ± 0.25°					
Marques *et al.*	2009	TLFB (30 days) DSM-IV C-DIS (Module R) AUDIT DRINC TRI	17.2 ± 17.22 (*n* = 120)	1st day – 8th Month	-	Whole blood	Total PEth	HPLC-ELSD 0.22 μM #**	0.43 ± 0.51	-	-	-	-	MCV, ALT, AST, GGT, %CDT, FAEE hair, ETG urine, ETS urine, ETG hair
			20.7 ± 25.5 (*n* = 243)						0.61 ± 0.61					
			29.55 ± 25.2 (*n* = 99)						1.45 ± 1.17					
Varga *et al.*	2000	-	-	-	Negative BrAC	Whole blood Erythrocytes MN leukocytes PMN leukocytes Plasma	Total PEth	HPLC-ELSD 0.22 μM #**	D1: 2.5 ± 0.9 (*n* = 6)	-	-	-	-	CDT, GGT
			150–300 (range)	Days: 1					D1: 5.1 ± 4.7 (*n* = 15)					
				3					D3: 3.9 ± 2.8° (*n* = 13)					
				5					D5: 2.5 ± 2.2° (*n* = 9)					
				7					D7: 2.4 ± 2.5° (*n* = 10)					

* Data are reported as Mean (M) ± Standard Deviation (SD), or Median (Me) with Interquartile Range (IQR) according to the type of statistical distribution;

& Mean;

$ Total range is reported; £ starting from the end of controlled alcohol administration;

# LOQ;

^ As supplied by the Authors;

° Calculated from reported data;

** Cut-off;

- = Not reported;

BAC = Blood Alcohol Concentration; BrAC = Breath Alcohol Concentration; TLFB = Time-Line Follow-Back; DSM-IV = Diagnostic and Statistical Manual of Mental Disorders IV; C-DIS = Computerized Diagnostic Interview Schedule; AUDIT = Alcohol Use Disorders Identification Test; DRINC = Drink Inventory of Consequences; TRI = Temptation and Restraint Inventory; TLC = Thin Layer Chromatography; LOQ = Limit of Quantification; HPLC = High Pressure Liquid Chromatography; PPV = Positive Predictive Value; NPV = Negative Predictive Value; LC = Liquid Chromatography; MS = Mass Spectrometry; ELSD = Electro-Light Scattering Detector; PEth = Phosphatidil-Ethanol; CDT = Carbohydrate Deficient Tranferrin; GGT = Gamma-Glutamyl Transpeptidase; MCV = Mean Corpuscolar Volume; AST = Aspartate Transaminase; ALT = Alanine Aminotransferase; GCDT = Gamma-CDT index(1.35 × ln CDT+0.8 × ln GGT); HRMS = High Resolution Mass Spectrometry; PMN = Polymorphonuclear cell; ETG = Ethyl-Glucuronide; ETS = Ethyl-Sulphate; FAEE = Fatty Acid Ethyl Esters; UETS = Urinary Ethyl Sulphate; UETG = Urinary Ethyl Glucuronide.
